# A microRNA profile of saliva and role of miR-375 in *Haemaphysalis longicornis* (Ixodida: Ixodidae)

**DOI:** 10.1186/s13071-019-3318-x

**Published:** 2019-02-01

**Authors:** Muhammad Irfan Malik, Mohsin Nawaz, Ibrahim A. Hassan, Houshuang Zhang, Haiyan Gong, Jie Cao, Yongzhi Zhou, Jinlin Zhou

**Affiliations:** Key Laboratory of Animal Parasitology of Ministry of Agriculture, Shanghai Veterinary Research Institute, Chinese Academy of Agricultural Sciences, Shanghai, 200241 China

**Keywords:** *Haemaphysalis longicornis*, Saliva, miRNAs, miR-375

## Abstract

**Background:**

Tick saliva contains many bioactive molecules that are involved in attachment to the host, blood-feeding and transmission of pathogens. MicroRNAs (miRNAs) are a class of short non-coding RNAs with a length of 19–24 nucleotides. They act as regulators of gene expression by binding to their target mRNA at the post-transcriptional level and control a variety of cellular functions, including regulation of growth, metabolism and development. The detection and characterizations of miRNAs from tick saliva may help explain the molecular mechanisms involved in the interaction between ticks, pathogens and hosts. They may also contribute to the discovery of vaccines, which can control ticks and the pathogens they transmit.

**Results:**

An RNA library was generated from the saliva of fed adult *Haemaphysalis longicornis* ticks, containing 17.4 million clean reads of 18–30 nucleotides. Overall, 319 known miRNAs and 1 novel miRNA were found. The 10 most abundantly expressed miRNAs present in tick saliva were miR-100_2, miR-315, miR-184_1, miR-100-5p_2, miR-5307, miR-184-3p_3, Let-7-5p_6, miR-71_5, miR-1-3p_6 and miR-10-5p_2. miR-375, one of the abundantly expressed, was subjected to quantitative real-time PCR analysis (qRT-PCR) in various tick developmental stages, as well as in different tissues isolated from adult ticks. The expression of miR-375 in different tick development stages was highest in unfed nymphs and lowest in the egg stage. In the tissues of adult ticks, miR-375 was most highly expressed in the salivary gland. To investigate the possible role of miR-375, Ant-375 was used to inhibit the miR-375. The treated group (Ant-375) had a reduced number of eggs (*t*_(10)_ = 2.652, *P* = 0.0242), eggs that were partially desiccated, and reduced egg hatchability (*t*_(10)_ = 2.272, *P* = 0.044) compared to Ms-Ant and the non-injected control.

**Conclusions:**

This is the first study to investigate the miRNA profile in tick saliva and the role of miR-375 in *H. longicornis*. The identification and characterization of miRNA in tick saliva may help to reveal the molecular mechanisms of interactions among ticks, pathogens and hosts, and suggest new vaccine strategies to control tick-borne diseases.

**Electronic supplementary material:**

The online version of this article (10.1186/s13071-019-3318-x) contains supplementary material, which is available to authorized users.

## Background

Ticks are obligatory blood-sucking ectoparasites involved in the transmission of a variety of disease agents, including bacteria, viruses and protozoa, to human and animals. Tick saliva not only assists in the feeding process but is also responsible for the transmission of pathogens to the host. In the tick-host interaction, saliva containing bioactive molecules affects a broad range of host physiological mechanisms, predominantly inhibiting host defense responses to promote tick-feeding [1, 2].

During the feeding process, ticks insert their hypostome and inject saliva into host skin as a response innate haemostatic system activates. Tick saliva counteracts both the hemostasis and defense mechanisms of the host through bioactive molecules, including anticlotting, antiplatelet aggregation, anti-inflammatory, vasodilatory and immunomodulatory factors [3]. The infectious pathogens are ingested along with the blood meal from an infected host. These pathogens impede the digestive epithelium in the midgut, invade the haemocoel, and finally infect the salivary gland through the salivary gland epithelium [4]. From the salivary gland, infectious agents can be transmitted to a new host *via* “saliva-assisted transmission” (SAT) as saliva is injected into a host during a new blood meal [5]. Therefore, the detection and characterization of bioactive molecules of tick saliva may help explain the molecular mechanisms of communication between ticks, pathogens and hosts.

MicroRNAs (miRNAs) are short non-coding RNAs ranging from 19 to 24 nucleotides in length. They act as regulators of gene expression by binding to their target mRNA at the post-transcriptional level *via* base pairing to target sites within the mRNA [6]. MicroRNAs are classified as pri-miRNAs, pre-miRNAs and mature miRNAs. RNA polymerase II transcribes encoded miRNAs in intergenic regions into long primary segments, which range from hundreds to thousands of nucleotides in length called primary miRNAs (pri-miRNAs) [7]. The pri-miRNAs, which normally comprise a single or several miRNAs as stem-loop, hairpin structures are cleaved into 70 nt sequences known as precursors (pre-miRNAs). Finally these pre-miRNAs are cleaved near their loops to generate a heteroduplex of two ~23 nt RNAs by the cytoplasmic RNase III enzyme Dicer, and these are then packed into a RISC complex [8]. The identification of miRNAs has increased prospects for better understanding the biology of many species. Several studies on tick saliva proteomics have been reported in *Amblyomma sculptum* [3], *Ixodes scapularis*, *Amblyomma americanum* [9] and *Haemaphysalis longicornis* [10]*.* Tick saliva miRNAs in *Ixodes ricinus* have been identified [11] but there are no reports of specific miRNA profiles related to tick saliva in *H. longicornis*. A previous study on the miRNA profile of the salivary gland in *H. longicornis* reported miR-375 as the most abundantly expressed in both unfed and fed adult ticks [12]. miR-375 is also expressed in human salivary glands [13, 14] and could play a role in the diagnosis and prognosis of human colon cancer.

The hard tick *H. longicornis*, transmits many infectious pathogens, including bovine theileriosis (caused by *Theileria* spp.), bovine babesiosis (caused by *Babesia ovata*), canine babesiosis (caused by *Babesia gibsoni*) and human rickettsiosis (caused by *Rickettsia japonica*) [15, 16]. *Haemaphysalis longicornis* is distributed mainly in East Asia and Australia. A greater understanding of its biology could lead to the development of more effective control measures.

## Results

### sRNA library from tick saliva

We constructed a small RNA (sRNA) library from the saliva of fed adult *H. longicornis*, using Hiseq technology [17, 18]. This generated 19,680,704 raw tags (Table 1), which contained different types of impurities. These included 5'-primer contaminants, no-insert tags, oversized insertion tags, low quality tags, poly A tags, small tags, and tags without a 3'-primer. All these contaminants were filtered according to the base quality value. We trimmed the adaptor sequence at the 3'-primer terminus, cleaned up 5' adaptor contaminants formed by ligation, and collected the RNAs and analyzed their size distribution.

**Table 1:** Summary of sequence data and alignment statistics of tags to reference genome

Sequence data and alignment statistics	Total tag counts
Summary of sequence data	
Raw tag count	19,680,704
Clean tag count	17,439,606
Percentage (%)	88.61
Alignment statistics of tags align to reference genome	
Total tag	17,439,606
Mapped tag	66,796
Percentage (%)	0.38

**Fig. 1:**
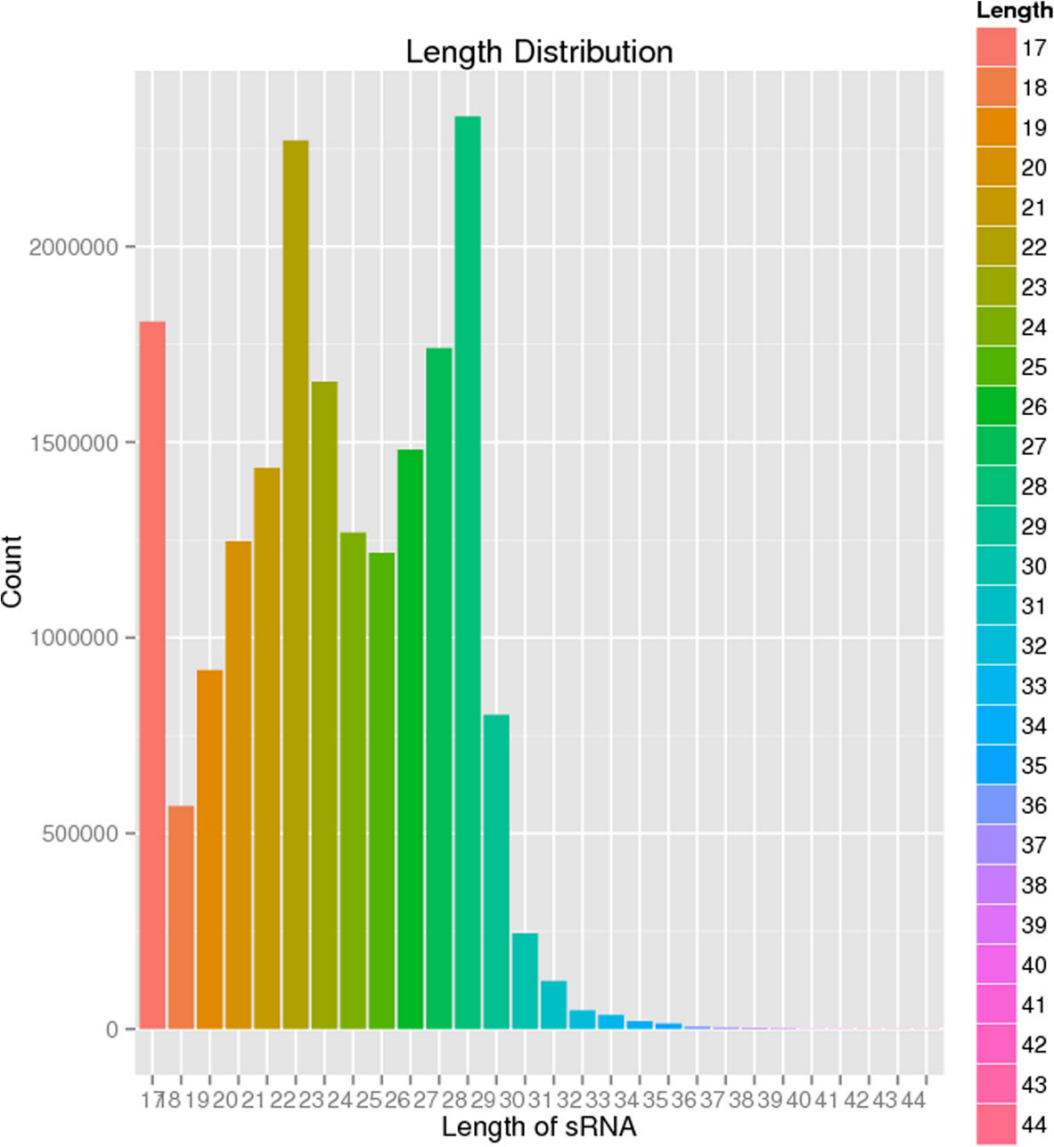
Length distribution of small RNA (sRNA). The X-axis shows the length of sRNA and the Y-axis shows the percentage of the number of sRNA with a specific length

**Fig. 2:**
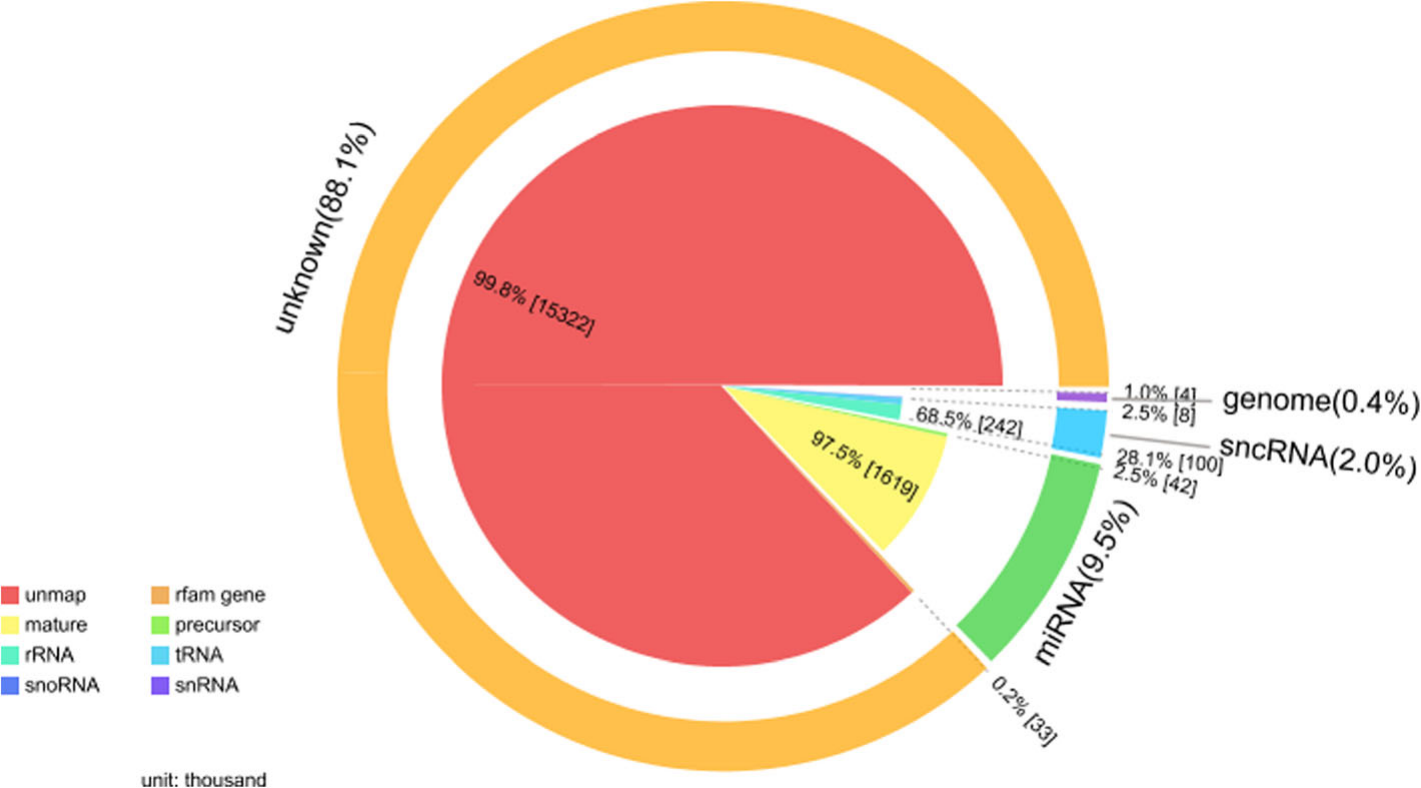
Proportion of different kinds of small RNA (sRNA). To make sure every unique small RNA mapped to only one annotation, we followed the following priority rule: miRNA > piRNA > snoRNA > Rfam > other RNA

**Fig. 3:**
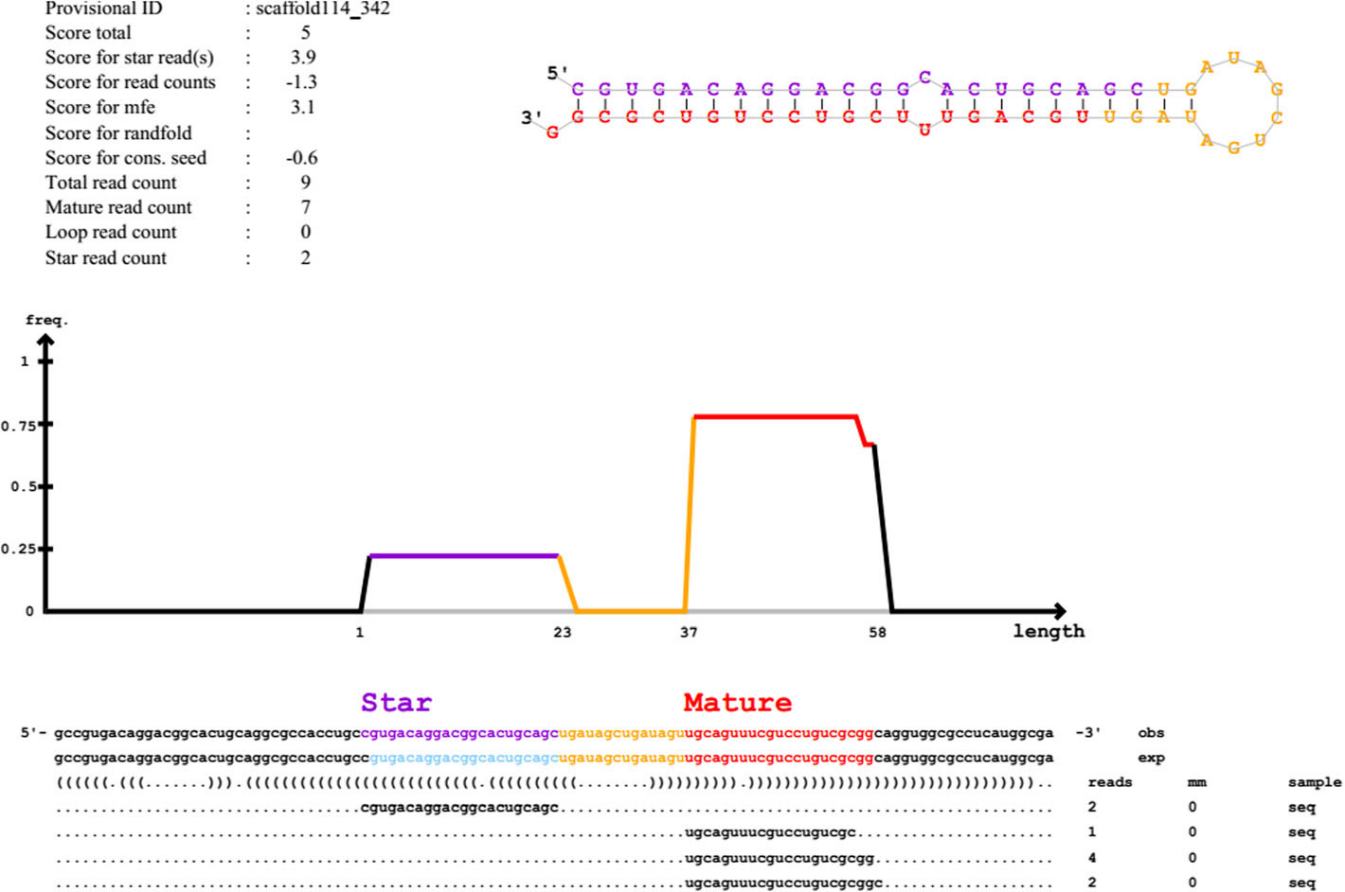
Secondary structure of candidate miRNA was predicted based on maximum free energy (MFE) structure and the sequence is marked with a red line

Deep sequencing yielded about 17,439,606 high-quality clean reads and 66,796 mapped tags reads (Table 1). The length distribution of total reads showed a unique bimodal division, which provides information about different types of small RNAs present in the sample, and it ranged between 18 nt and 30 nt (Fig. 1). One peak around 22 nt represented mature miRNA and a second broader peak around 28 nt constituted a proportion of small RNA. A major portion of the sRNA library was unknown (88.1%) and unmapped (99.8%) because of the unavailability of the whole genome sequence of *H. longicornis*. miRNAs (9.5%) account for a major portion and are comprised of mature miRNA (97.5%) and precursor miRNA (2.5%). The remaining part includes genome (0.4%) and sncRNA (2%), rRNA (68.5%) and tRNA (2.5%) (Fig. 2). The analysis of *H. longicornis* tick saliva produced 319 known miRNAs, 1 novel miRNA, 76 precursor and 228 SnoRNA (Additional file 1). The miRNAs miR-92, miR-263, miR-9, miR-25 and miR-9 had more than ten genomic copies (subtypes), 135 miRNAs had less than ten and 37 miRNAs had only a single genomic copy. The top ten abundantly expressed miRNAs in tick saliva and comparison with the top 10 abundantly expressed miRNAs in the salivary gland of fed adult *H. longicornis* ticks [12] are shown in Table 2. The general structure of the precursor of the predicted miRNA with a stem loop mature sequence is shown in Fig. 3.

**Table 2:** Ten most abundant miRNAs in the saliva and salivary gland of fed adult ticks

Saliva	Count	Salivary gland	Count
miR-100_2	415,333	miR-1	445,787
miR-315	309,466	miR-375	376,709
miR-184_1	275,170	miR-184	154,004
miR-100-5p_2	140,614	miR-263a	79,829
miR-5307	119,499	Bantam	74,385
miR-184-3p_3	109,545	miR-739	53,501
Let-7-5p_6	33,924	miR-317	39,473
miR-71_5	32,787	Let-7	25,833
miR-1-3p_6	22,022	miR-275	15,620
miR-10-5p_2	10,100	miR-183	868

### Expression profile of miR-375 in various developmental stages and tissues

**Fig. 4:**
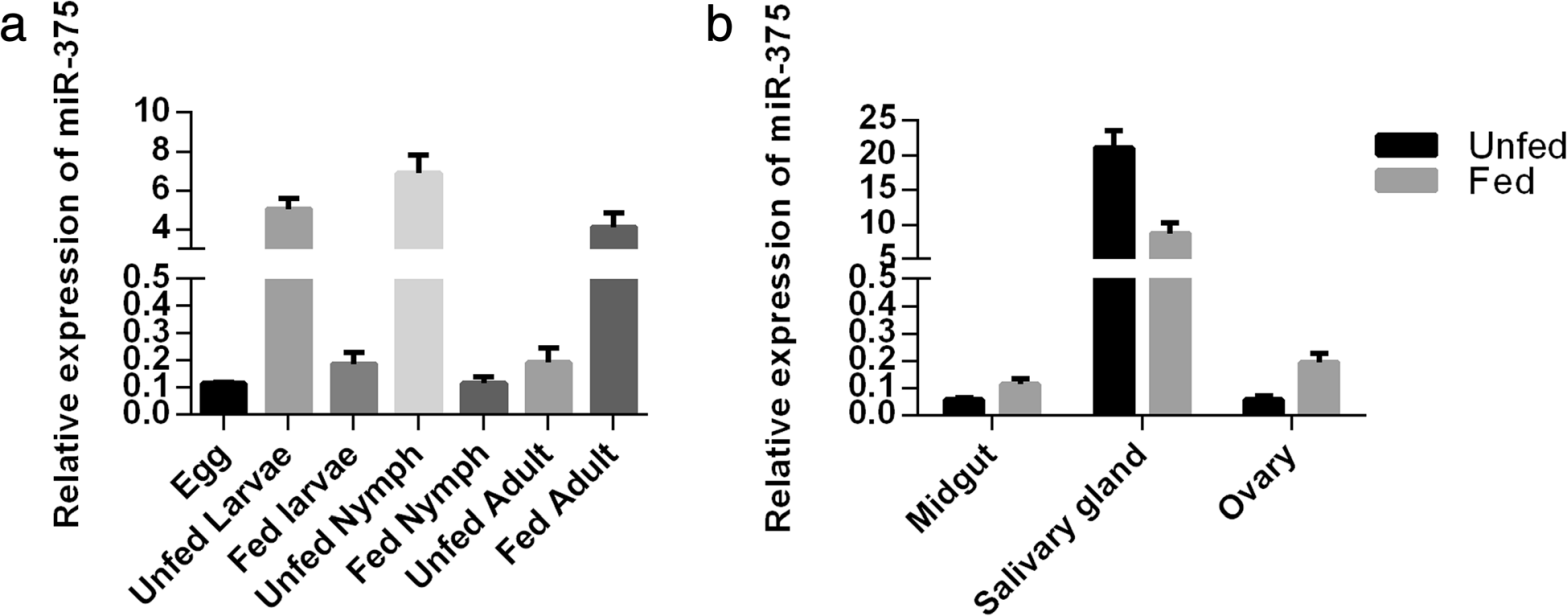
Relative expression of miR-375 in different stages of life-cycle (**a**) and in different tissues (**b**) of unfed and fed adult *H. longicornis* tick. Data represent three biological replicates with three technical replicates and are shown as mean ± SEM

miR-375 was highly expressed in the salivary gland and saliva of *H. longicornis*. To determine the possible biological function of miR-375, real-time PCR was used to measure the expression levels in different developmental stages (egg, unfed larva, fed larva, unfed nymph, fed nymph, unfed adult and fed adult) and various tissues collected from unfed and fed adult ticks (midgut, ovary and salivary glands). Expression was significantly lower in eggs, fed (larva, nymph) and unfed adults. Expression was higher in unfed (larva, nymph) and fed adults and highest in unfed nymphs (Fig. 4a). The tissue expression of mature miR-375 was highest in the salivary gland and lower in other tissues (ovary, midgut) in unfed and fed adult ticks (Fig. 4b). The expression of miR-375 in the salivary gland decreased in fed adults compared to unfed adult ticks.

### Silencing of miRNA-375 affects egg production and hatchability

**Fig. 5:**
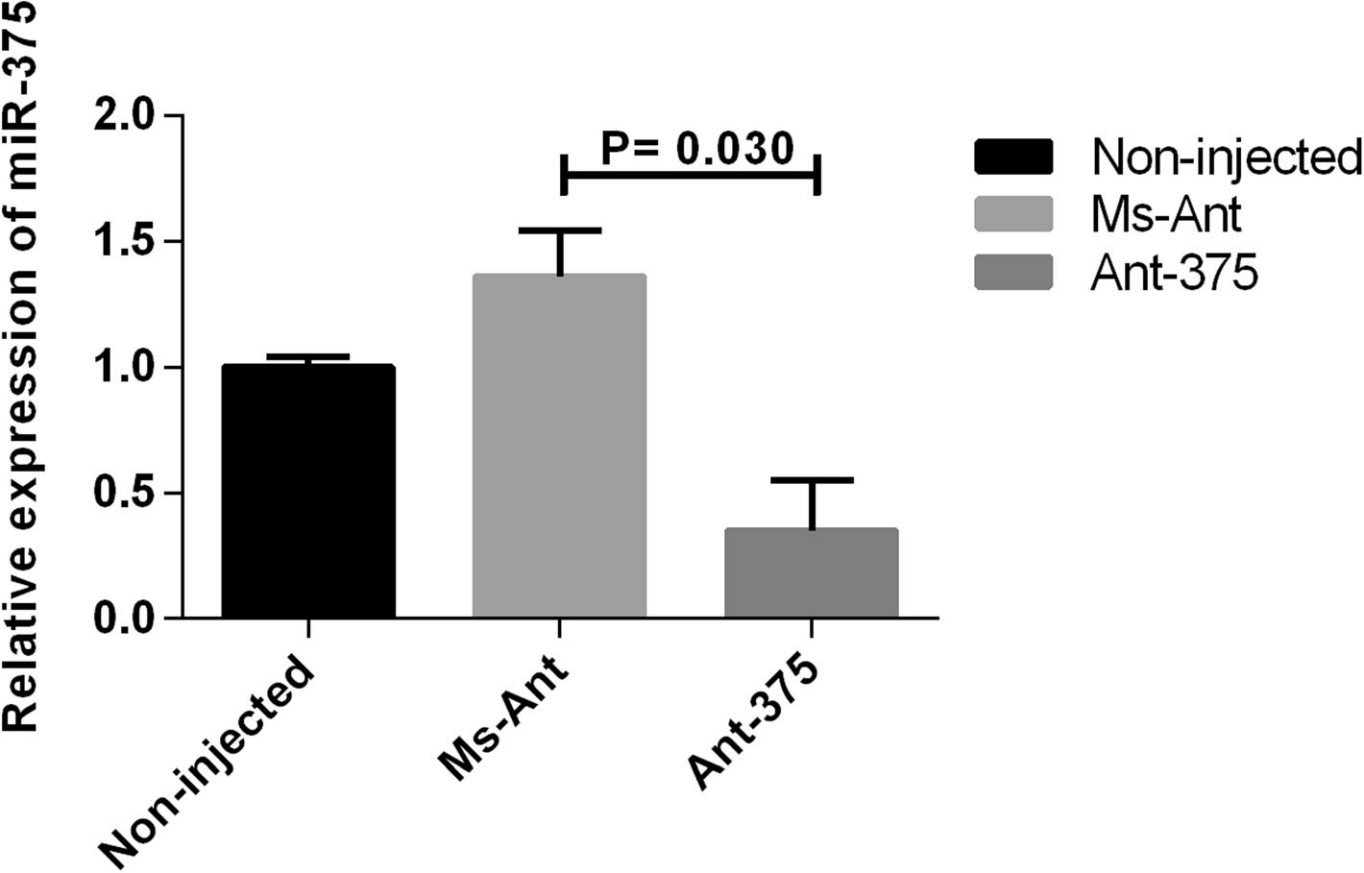
The miR-375 inhibition by Ant-375. Data represent three biological replicates with three technical replicates and are shown as mean ± SEM

To explore the possible roles of miR-375 in adult female *H. longicornis*, the sequence-specific antagomir (Ant-375) was used to knock down the miR-375. Each unfed tick was microinjected with 0.5 μl (10 μM) of Ant-375 and Ms-Ant and ticks were incubated at 25 °C, 92% RH for 24 h and then placed on a rabbit for attachment and blood-feeding. Quantitative real-time PCR was performed at 4 days post-feeding to evaluate the silencing of miR-375. The results showed that relative expression of miR-375 was down regulated to 32.4% in Ant-375 compared to the Ms-Ant control and non-injected ticks (Fig. 5; *t*_(4)_ = 3.296, *P* = 0.030).

**Fig. 6:**
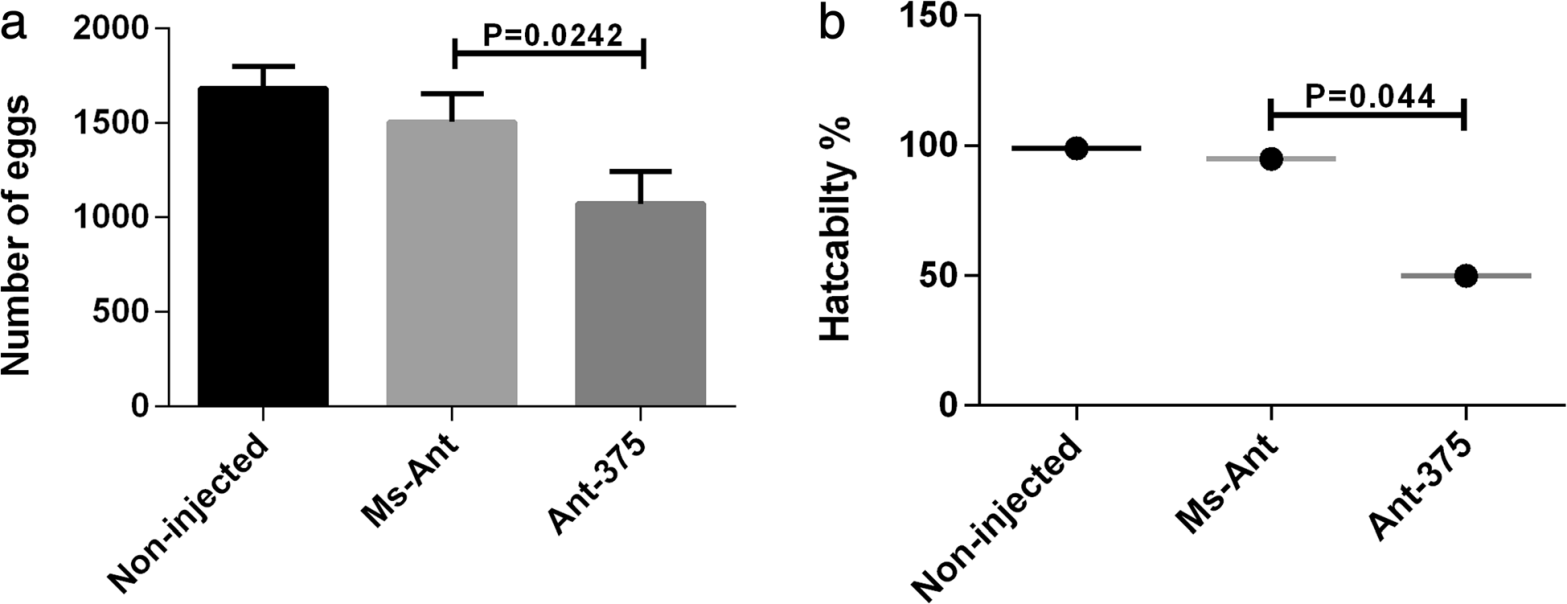
Silencing of miR-375 affects the egg number (**a**) and hatching in Ant-375, Ms-Ant and non-injected ticks (**b**)

**Fig. 7:**
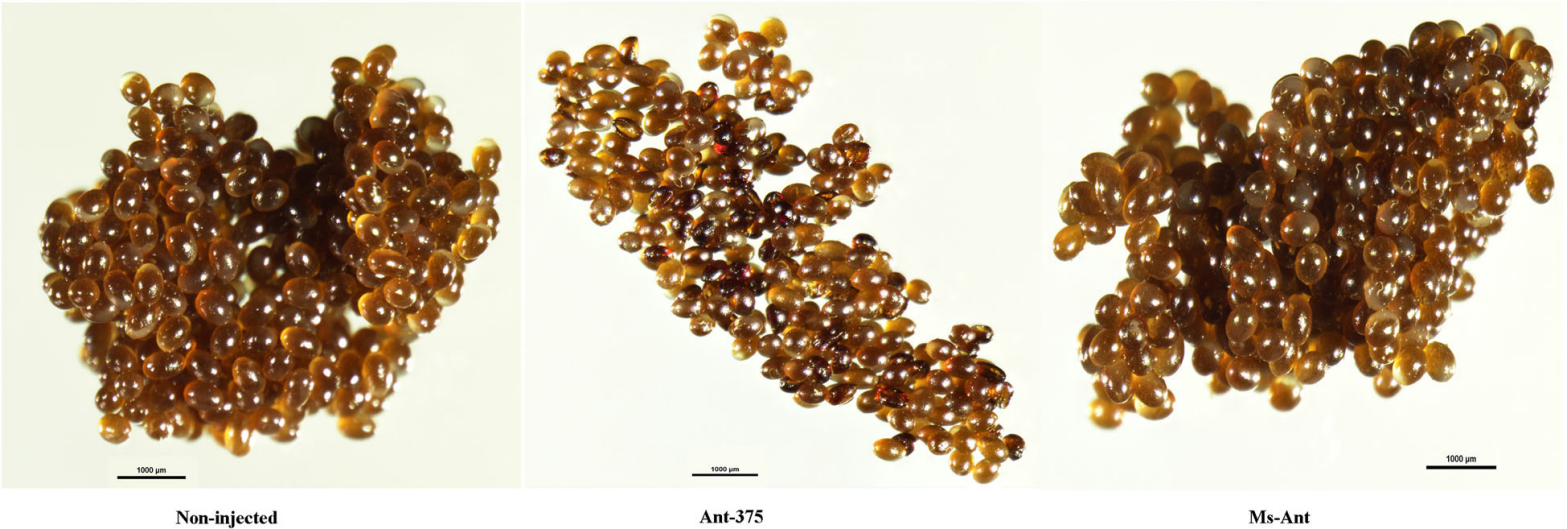
miR-375 inhibition effects on egg development in Ant-375 compared to non-injected and Ms-Ant control

Different biological parameters (attachment rate, engorged body weight and tissue appearance) were also observed and analyzed in each group after silencing of miR-375. Attachment rate, engorged body weight and blood-feeding between miR-375 treated and control groups were similar. The tissues (salivary glands, midguts, ovaries) were also observed after silencing but there was no obvious change in their appearance. However, egg production and hatchability were significantly reduced after silencing of miR-375. Approximately 6 days after engorgement, most *H. longicornis* females began to lay eggs. The average number of eggs from Ant-375 ticks was 1072, while the Ms-Ant control and blank control produced 1683 and 1570, respectively (*t*_(10)_ = 2.652, *P* = 0.0242; Fig. 6a). The mean length of the egg laying period was 28.6 ± 0.748 days for Ant-375 ticks and 27.1 ± 0.860 and 26.0 ± 0.707 days for Ms-Ant and non-injected ticks, respectively (Table 3). Egg hatchability was 99% in non-injected ticks, 95% in the Ms-Ant control and 50% in Ant-375 ticks (*t*_(10 )_ = 2.272, *P* = 0.044; Fig. 6b). The eggs from the Ant-375 group became partially desiccated compared to eggs in the control groups (Fig. 7).

**Table 3:** Feeding periods and oviposition in different groups

Group	Feeding period (days)	Oviposition (days)
Non-injected	6.16	26.0 ± 0.7078
Ms-Ant	5.75	27.1 ± 0.860
Ant-375	5.37	28.6 ± 0.748*

*P < 0.05; *(t*_(10)_ *=* 1.606*, P =* 0.1393)

## Discussion

Tick saliva contains a variety of bioactive molecules, including miRNAs, which are involved in attachment to the host, blood-feeding and transmission of tick-borne pathogens. Micro-RNAs act as regulators of gene expression by binding to their target mRNA at the post-transcriptional level and control different functions in cells, including regulation of growth, metabolism, differentiation and development [19]. There are a few studies on the expression and other functions of miRNAs in ticks. The tick saliva proteomics in *H. longicornis* [10] has been examined, and there is a report on salivary miRNA in *Ixodes ricinus* [11]. However, there is no information on tick saliva miRNA in *H. longicornis*. We present the first data on the saliva-specific miRNA profile in *H. longicornis*. *Ixodes scapularis* was used as a reference tick genome.

We used Hiseq technology to obtain 19.6 million raw tags and 17.4 million clean tags from the saliva of fed adult ticks (Table 1). A previous report on salivary gland miRNAs from fed adult ticks yielded 14.8 million reads, which is lower than the 17.4 million reads in our study [12]. The major portion of the small RNA library is unknown (88.1%) and unmapped (99.8%) because the whole-genome sequence of *H. longicornis* is unavailable. We obtained 319 known miRNAs and one novel miRNA (Additional file 1). We also compared the top ten abundantly expressed miRNAs from fed adult tick saliva with miRNAs from the salivary gland of fed adult *H. longicornis* ticks from a previous study [12]*.* The result was different in abundantly expressed tick saliva miRNAs wherein only three miRNAs families (miR-1, miR-184 and Let-7) were conserved (Table 2). These highly expressed miRNAs may be evolutionarily conserved in *H. longicornis*. The results also showed that expression of the miRNA significantly changes from the salivary gland to the saliva of fed adult ticks.

A previous miRNA profile of the *H. longicornis* salivary gland showed miRNA-375 as one of the most highly expressed miRNAs [12]. miRNA-375 is also expressed in *I. scapularis* [20] and human salivary glands [13, 14]. Exosome-carried miR-375 inhibits cell progression and the dissemination of colon cancer in humans [21] and may be valuable for the prognosis and diagnosis of cancer in humans [22]. The miR-375 is one of the 20 most highly expressed miRNAs in tick saliva and currently there is no study on miR-375 in ticks. We therefore selected and analyzed the expression of miR-375 in different stages (egg, unfed larva, fed larva, unfed nymph, fed nymph, unfed adult and fed adult) within the tick life-cycle. Expression was highest in unfed nymphs and lowest in the egg stage (Fig. 4a). High expression in unfed nymphs suggests that miR-375 has a role in tick development and maturation. The tissue distribution of miR-375 showed that it was highly expressed in the salivary gland. Expression was highest in unfed compared to fed adult tick salivary glands and there was low expression in other tissues (Fig. 4b). To explore the possible function of miR-375 in *H. longicornis*, Antagomir-375 (Ant-375) was used to inhibit the miR-375 (Fig. 5). Inhibition of miR-375 affected egg production (Fig. 6a) and hatchability (Fig. 6b) in contrast to the negative control and non-injected blank ticks. Ticks of the different experimental groups were dissected and internal tissues (salivary glands, midguts, ovaries) were also examined after silencing but no obvious morphologic changes were observed. The eggs were also monitored during oviposition in the three groups. Eggs of Ant-375 group became partially desiccated (Fig. 7) and had reduced hatchability. Currently there is no specific study on miR-375 in ticks, but recent research on miR-275 in *H. longicornis* [23] and the mosquito *Aedes aegypti* [24] showed that silencing resulted in egg number reduction and development inhibition, respectively. Other studies have demonstrated that oviposition and egg hatching rate can be negatively affected after immunization or silencing [25, 26]. In ticks, yolk protein (vitellin) is synthesized in the form of precursor vitellogenin in the fat body, secreted into hemolymph and taken up by the developing oocyte during oogenesis. Oocyte growth depends on the uptake of vitellogenin. The relationship or interaction between miR-375 and vitellogenin needs further investigation. Our results suggest that miR-375 could have a role in oviposition and egg hatching of *H. longicornis*. Further functional characterization is required to verify miR-375 and other miRNAs and their exact target genes in ticks. Future experiments will include detailed analysis of each miRNA and their target prediction, validation and mechanism of action in the invertebrate vector.

## Conclusions

This is the first study to investigate the miRNAs in saliva and the role of miR-375 in *Haemaphysalis longicornis* tick. This identification and characterization of miRNA in tick saliva may provide insights into the molecular interactions between ticks, pathogens and vertebrate hosts, and may contribute to the development of vaccines that will reduce tick-borne diseases.

## Methods

### Tick rearing and feeding

*Haemaphysalis longicornis* ticks were maintained in our laboratory at the Chinese Academy of Agricultural Sciences Shanghai, China [27]. Approximately 300 ticks were confined in cloth bags and fed on a New Zealand rabbit ear until they were semi-engorged. The ticks were collected 4 days after feeding, washed with 75% ethanol and used for saliva collection.

### Saliva collection

The 4 days post-feeding ticks were immediately washed with 75% ethanol and fixed ventral side up on a glass slide by scotch tape. The saliva was collected from the ticks using a procedure described by Patton et al. [28]. Briefly, the ticks were micro-injected with 0.5 μl of 10% pilocarpine in the genital pore and held for 1–2 min until salivation started. The saliva was then collected from the tick hypostome by inserting a pipette tip containing a small amount of PBS (phosphate buffered saline). Approximately 1 to 1.5 μl saliva was obtained from each tick. The collected saliva was put in TRIzoI reagent (Invitrogen, Carlsbad, CA, USA) and placed on ice. The saliva was repeatedly collected from each tick for 1–2 h and the final volume was stored at -80 °C.

### Sample collection at different developmental stages and tissue dissection

Samples were collected at different development stages (egg, unfed and fed larvae, nymph and adult) of ticks. The tissue samples (salivary glands, midguts and ovaries) were dissected from unfed and 4 days semi-engorged adult ticks. Tick dissection was performed as described by Gao et al*.* [29]. Briefly, unfed and partially fed ticks were submerged in ice-cold phosphate-buffered saline (autoclaved; pH 7.4) under a dissection light microscope and held with a pair of soft-tissue forceps. The dorsal cuticle was removed and the various tissues (salivary glands, midgut and ovary) were separated using 18-gauge needles. After dissection, these tissues were washed with PBS and put into microcentrifuge tubes, and preserved at -80 °C.

### Total RNA isolation and real-time PCR

The total RNA was extracted from saliva, different developmental stages and various tissues (salivary gland, midgut and ovary) of ticks using TRIzoI Reagent (Invitrogen) according to the

manufacturer’s protocol. The purity and integrity of the total RNA were determined by standard agarose gel electrophoresis, while the concentration was measured using a BioPhotometer (GE, Fairfield, CT, USA). The purified total RNA was stored at -80 °C until deep sequencing or quantitative (q) RT-PCR.

The cDNA for miRNA was transcribed with a miScript II RT kit (Qiagen, Hilden, Germany) using Hiflex buffer. The forward primer (5'-TTT GTT CGT TCG GCT CGA GTT AA-3') and universal reverse primer included in the miScript SYBR Green PCR kit were used to amplify miR-375, and tick elongation factor 1α (ELF1A) was used as the internal control. Expression of miR-375 was analyzed by real-time PCR performed on an Applied Biosystems 7500 (Waltham, Massachusetts, USA) using miScript SYBR Green PCR kit (Qiagen) according to the manufacturer’s protocol. All reactions were performed in triplicate. The relative expression for miRNA was measured using 2^-∆Ct^ method.

### Feeding periods and oviposition

The feeding periods for each group were calculated using the formula: number of ticks engorged × feeding days/total number of ticks. Oviposition was measured as follows: average of number of days (start laying-stop laying eggs)/total number of egg laying ticks) as per the procedure used by Hadi & Adventini [30].

### Antagomir synthesis and application

Antagomir are miRNA-specific antisense oligonucleotides that we used for the inhibition of miR-375. The missense antagomir (Ms-Ant) was used as a negative control of antagomir and was purchased from GenePharma (Shanghai, China; http://www.genepharma.com). Non-injected ticks were used as the blank control. Unfed adult female *H. longicornis* ticks were microinjected through the joint between the IV coxa and the sternal plate with antagomir and Ms-Ant at a dose of 0.5 μl (10 μM). Each group contained 38 ticks and maintained in an incubator at 25 °C with 92% RH for 24 h and then infested on a rabbit for blood-feeding.

### Statistical analysis

All data were analyzed with GraphPad Prism 6 using Student’s t-test, and the results are shown as mean ± SEM (*P* < 0.05 was considered as significant).

## Additional file

Additional file 1: Tick saliva miRNA. (XLSX 47 kb)

## Abbreviations

Ant: Antagomir
miRNA: Micro RNA
Ms-Ant: Missense antagomir
PBS: Phosphate buffered saline
RH: Relative humidity
rRNA: Ribosomal RNA
snoRNA: Small nucleolar RNA
snRNA: Small nuclear RNA
sRNA: Small RNA
tRNA: Transfer RNA
